# Inspectors’ ethical challenges in health care regulation: a pilot study

**DOI:** 10.1007/s11019-016-9736-z

**Published:** 2017-01-27

**Authors:** W. Seekles, G. Widdershoven, P. Robben, G. van Dalfsen, B. Molewijk

**Affiliations:** 1 Medical Humanities, VU University Medical Centre (EMGO+), Van der Boechorststraat 7, 1081 BT Amsterdam, The Netherlands; 2 The Health Care Inspectorate (IGZ), Stadsplateau 1, 3521 AZ Utrecht, The Netherlands; 3 University of Humanistic Studies, Kromme Nieuwegracht 29, 3512 HD Utrecht, The Netherlands; 4 Centre for Medical Ethics, HELSAM, University of Oslo, Forskningsveien 3A, 0373 Oslo, Norway

**Keywords:** Ethical challenges, Moral questions, Health care inspectorate, Health care regulation, Ethics support, Moral case deliberation

## Abstract

There is an increasing body of research on what kind of ethical challenges health care professionals experience regarding the quality of care. In the Netherlands the Dutch Health Care Inspectorate is responsible for monitoring and regulating the quality of health care. No research exists on what kind of ethical challenges inspectors experience during the regulation process itself. In a pilot study we used moral case deliberation as method in order to reflect upon inspectors’ ethical challenges. The objective of this paper is to give an overview of the ethical challenges which health care inspectors encounter in their daily work. A thematic qualitative analysis was performed on cases (*n* = 69) that were collected from health care inspectors in a moral case deliberation pilot study. Eight themes were identified in health care regulation. These can be divided in two categories: *work content* and *internal collaboration*. The work of the health care inspectorate is morally loaded and our recommendation is that some form of ethics support is provided for health care inspectors.

## Background

There is an increasing body of research on the field of ethical challenges in healthcare^1^ e.g. in the care of patients with ALS (Seitzer et al. 2016), in elderly and dementia care (Hasselkus 1997; Van der Dam 2011; Bolmsjö et al. 2006), in long-term care (Elander et al. 1993), and in (community) mental healthcare (Liégeois and Van Audenhove 2005; Molewijk et al. 2008a, b, 2015). Most ethical challenges are related to quality of care and quality of the organization of health care institutions. The Dutch Health Care Inspectorate (IGZ) monitors and regulates the quality of healthcare in the Netherlands. Like health care professionals, inspectors focus on quality of care. This poses the question as to whether inspectors of the Health Care Inspectorate do not also experience ethical challenges in monitoring the quality of care and, if so, whether these ethical challenges are comparable to the ethical challenges of health care professionals in general. There might be differences, since inspectors access quality of care from a different angle, i.e. more formal and regulative. We therefore pose the question as to what kind of ethical challenges do the inspectors of the Dutch Health Care Inspectorate experience when supervising the quality of care?

The IGZ is the body appointed by the government and operates as an independent part of the Ministry of Health, Welfare and Sports (VWS). Its position, tasks and responsibility is comparable to the Care Quality Commission (CQC) in the United Kingdom and the health care inspectorates in the Scandinavian countries. Inspectors from regulating bodies such as the IGZ encounter difficult decisions every day. Their work takes place in an arena with diverse and high expectations of citizens, professionals, politicians and directors (WRR 2013). This complex work environment often leads to dilemmas in regulation such as confidentiality versus transparency and proximity versus distance (Robben 2010). In the daily practice of regulation these difficult situations or dilemmas are often seen or approached as technical questions that can be solved on the basis of practical experience, new knowledge based on research, law and legislation, or rules and guidelines (OECD 2014). However, these difficult situations or dilemmas also have a *moral* component for the individual inspector, their managers and even the IGZ as an organization, and the nature of these moral components has not in our view been sufficiently explored. Until now, no systematic attention has been given to the moral dimension of health care regulation and how to deal with the associated ethical challenges in daily practice.

This is in contrast with the still growing attention for the ethical challenges health care professionals experience in health care (Molewijk et al. 2016). During the last decade, various forms of clinical ethical support arose in many health care institutions in Europe in order to better deal with the inherent moral dimension of the healthcare profession (Hurst et al. 2007; Førde et al. 2008; Schildmann et al. 2010; Slowther et al. 2011; Dauwerse et al. 2014a, b). One of these clinical ethics support forms is moral case deliberation (MCD) (Molewijk et al. 2008c). Earlier evaluation research has demonstrated that MCD contributes to the moral competency of health care professionals (Abma et al. 2009; Molewijk et al. 2008a, b; Van der Dam et al. 2013; Weidema et al. 2013; Svantesson et al. 2014). In order to explore the ethical challenges IGZ inspectors encounter in their work and to investigate whether moral case deliberation, could be employed as a valuable tool supporting inspectors in the ethical challenges they face, we conducted an explorative pilot study with(in) the IGZ. The choice of using moral case deliberation in the regulating body on the quality and safety in healthcare is motivated by the fact that the method is widely used and positively evaluated in health care (Dauwerse et al. 2013) and also in line with the recommendation of the Dutch ministry of health (VWS) (VWS 2007) and the Dutch institute for accreditation in health care (NIAZ 2013) to organize structural attention for ethics and ethics support, amongst which MCD, in healthcare settings.

## Moral case deliberation

Moral case deliberation (MCD) is a systematic dialogue, led by a trained facilitator, in which professionals reflect upon a concrete case from daily practice (Molewijk et al. 2008c; Dauwerse et al. 2014a; Stolper et al. 2015). Within MCD, several conversation methods are use in order to structure the moral inquiry (Steinkamp and Gordijn 2003; Molewijk et al. 2008c). A dialogue as a work form is essential because there are no universal criteria to determine what is morally right. It is, however, not the aim of a dialogue to determine responsibility or fault but rather to bring to light different perspectives that can shed understanding of the moral challenges of that specific situation (Widdershoven and Molewijk 2010). Research demonstrates that MCD enables professionals to better deal with moral issues, better learn to analyse and reflect, better work together and enhances professionalism and professional accountability (Molewijk et al. 2008a, b). MCD is often used for the implementation of culture-change or professionalization movements (Dauwerse et al. 2013; Weidema et al. 2015). A recurring MCD offers stakeholders the possibility to make their normative expectations and notions about what makes their practice good or bad explicit and to engage in a conversation about these expectations and notions. Dialogue provides an opportunity to revitalize the practice; to discover by what axioms and assumptions one is guided; to place question marks; to motivate change in daily practice, and to highlight improvements, which could be made. Together these actions may lead to increase professionalism.

In this pilot study, the dilemma method for MCD is introduced in the context of the Dutch Health Care Inspectorate in order to support employees in dealing with moral questions in their profession (Molewijk and Ahlzen 2011; Stolper et al. 2016). The objective of this paper is to give an overview of the ethical challenges which health care inspectors encounter in their daily work.

## Methods

### Setting and participants

The present pilot study was carried out at the Dutch Health Care Inspectorate (IGZ). A total of ten primary process employees (senior inspectors and reporting centre employees) were included on voluntary basis. These participants participated in one MCD-group that came together during eight MCD sessions.

### Design

This pilot study is explorative and qualitative in nature. A previous paper on this pilot described the explorative qualitative analysis of the evaluation of MCD at the IGZ. A more detailed description of all used methods and the evaluation results in this pilot study are described elsewhere (Seekles et al. 2016). The current paper contains a thematic qualitative analysis of the content (casuistry) sent in every week before a MCD by the MCD participants. All participants were informed on the objectives of the pilot study in advance and gave informed consent before participating in the study. The presented cases and MCD reports are only shared with MCD participants and the researchers that were present during de MCD. The data (i.e. cases) are only accessible to the researchers. All presented casuistry is anonymous and read and approved by all the participants (member-check). The referral to males and females in the casuistry is randomly interspersed.

### Recruitment

Participants for the pilot study (and MCD series) were recruited via the intranet of the IGZ. Eligible participants could volunteer for participating in the pilot study. A total of 18 primary process employees registered and they were invited to an information meeting. During this meeting, additional information on the pilot study in general and on MCD in specific was given. A number of participants were excluded because they lacked time for the scheduled MCDs. Ten of the eighteen participants were included for the pilot study. One MCD participant left the pilot due to a new workload.

### Moral case deliberation

A series of eight moral case deliberations was organized between December 2012 and July 2013 (approximately one meeting a month). Every MCD (dilemma method) was scheduled for 90 min at the IGZ headquarters. The dilemma method consists of ten steps: introduction, presentation of the case, formulation of the dilemma, asking factual questions in other to replace oneself in the situation of the case-owner, collecting actual values and norms of the involved stakeholders, brainstorm on possible alternatives, formulating individual answers to the moral dilemma including argumentation and to deal with the inherent loss of the dilemma, investigation of the similarities and differences among the participants, conclusions and actions, evaluation and follow-up. Authors BM and GvD alternately acted as facilitators in the MCDs. Every session was audio taped (yet not transcribed). After each MCD, author WS wrote a confidential report that was distributed among the participants in order to have a final member-check.

### Ethical challenges

To explore what ethical issues arise in health care regulation, we asked all participants to send in work-related casuistry before every MCD. The instruction the participants received was to send in a brief and concrete description of a work-related case in which they experienced a moral dilemma or question. These descriptions contained personally experienced moral dilemmas combined with the formulation of a moral question. During each MCD session, one of the presented cases was chosen and discussed. In MCD these cases were translated into a moral dilemma with the following format: ‘Should I do behaviour A or behaviour B?’^2^ The related moral question often dealt with a more abstract conceptual question, which was related to the behaviour that was mentioned within the moral dilemma. All cases were collected and analysed by the researchers. In total, this resulted in 9 (participants) × 8 (MCD) = 72 cases. However, in a few occasions one of the participants found it difficult to identify a moral issue or lacked time to send one in, therefore a total of 69 cases were collected during this pilot study.

### Thematic qualitative analysis

The casuistry that was sent in by the participants of the MCD sessions is analysed by means of a table with *topics*, *moral question* and *themes*. All casuistry is analysed and categorized by minimal two independent researchers (WS, BM and GW). Initial analysing was performed in line with quality criteria described in the literature: remaining open, staying close to the data and keeping codes simple and precise (Mertens 2010). The authors individually constructed short summary descriptions, compared data, and then involved other research team members in the coding. We discussed differences in interpretation among WS, BM and GW. After consensus about the coding, the team went back to the case descriptions in order to check our summary codes.

## Results

The 69 cases sent in were described as concrete daily practical situations including feelings about these situations (hence not in terms of a theme or formulated in a dilemma with colliding values). In the analysis the collected cases are categorized by subject and moral questions. After analysing the cases, we identified eight themes. Table 1 3 gives an overview of the moral themes and the number of cases per theme. All 69 cases were categorized in at least one of the themes presented in Table 1. A total of 24 cases fall in two themes and one case in three themes. All eight moral themes in health care regulation are explained below and illustrated with an example from one of the cases. Conflicting values and norms are mentioned when they were discussed in one of the deliberations or when the respondent mentioned these in the description of the case.

**Table 1:** Number of cases per theme

Theme	Number of cases	Number of cases in more then one theme
How should we relate to others?	30	20
How should we cooperate with each other within the IGZ?	23	11
Intensified supervision or equity?	13	4
Is it allowed to give substantive judgements on care/profession?	6	2
What is an appropriate role of the inspector (IGZ) in a conflict between other parties?	6	2
What are the boundaries of the professional responsibility?	6	4
Should we always adjust to the new and stricter IGZ policy?	6	5
If and when do we have to share information?	5	4

### Theme 1: How should we relate to others?

The question as to how we relate to others has raised moral questions in health care regulation. In total, 30 of the 69 cases are categorized under the theme ‘how should we relate to others’. Within this theme, the cases concern the relationship of the inspector (or IGZ) with third parties. Third parties in the cases were citizens, politics, other inspectorates, (health care) professionals, (board of directors of) and health care institutions.^4^ An example of a case:

“An anonymous reporter has sent a letter to the minister of Welfare, Health and Sports to (once again) draw attention to the misconduct of a specific health care organization”. The ministry asks IGZ to investigate this, but the IGZ doubts the necessity of this inspection. This case raised the following moral question: “Should I, when there is no evidence of wrongdoing, nevertheless conduct an investigation because it is asked by the ministry?”

The case clearly shows the dilemma of the inspector where the inspector is faced with choosing a specific side; that of himself individually, the professional or the organisation. In a few other cases the question is if certain actions are needed or not and the effect they might have on others. Do you have to give certain information? May the inspector ask everything during a regulation? How do I treat incomplete reports of anxious citizens? The complex environment in which the inspectorate operates causes moral questions regarding relationships with others.

### Theme 2: How should we cooperate with each other within the IGZ?

As mentioned in the description of the previous theme, the inspector regularly encounters questions regarding relationships with others. A total of 23 cases are categorized in the theme internal cooperation. The moral questions in these cases often are related to addressing a colleague or manager. When and how do you express your own opinion or ideas? Or when and how do you tell your manager that you disagree?

This can be illustrated by the following case:

“A group of inspectors discusses a notification of a serious incident in health care in their weekly meeting. A health care organisation reports that one of their employees acted improperly, but research of the LMO^5^ shows that this information is incorrect. However, the LMO seems to ignore this new information and wants to proceed with an acute disciplinary complaint”. The moral question of the inspector was: “Should I try to convince my colleagues that an acute disciplinary complaint against the involved employee is not appropriate or should I follow the LMO in their decision?”.

The inspector thinks that the individual employee of the organisation has been treated too harshly. The other dilemma that plays a role in this case according to the inspector is imposing measures versus the quality of care. Conflicting values according to the participants were amongst others: integrity (i.e. being able to handle as I think I should), reliability (i.e. IGZ should be reliable), objectivity (i.e. that the judgment is transparent) and loyalty (i.e. towards your colleagues but also towards yourself).

Other similar moral questions with respect to internal cooperation from other cases were: “Do I have to make an issue of my principles or do I keep silent?”, “Do I have to say to my colleague that I don’t want to address the organisation on behalf of IGZ because I have a different opinion then my colleague?”, “Must I, in an evaluation with an IGZ-colleague, accept his way of functioning or should I discuss this with him?” or “Do I have to do what my manager tells me to do?”. In this last questions important conflicting values, according to case owner were: trust versus professionalism and openness (i.e. address each other). Most cases are about íf you have to/must address something to a colleague or manager or not or if you may/have to/must give your opinion if it differs from others or the IGZ policy.

### Theme 3: Intensified supervision or equity?

The balance between intensified supervision and equity regularly also raises several moral questions. Thirteen of the 69 of the cases are categorized in this third theme. The ethical dilemmas with respect to this theme are about making the right decision between intensified supervision when, on the one hand, a health care organisation does not meet the standards and on the other hand to take into account mitigating circumstances and striving for equity. An example of a case:

“A young professional, three years after his graduation, is manager in a chaotic health care organisation. “She was thrown to the wolves” [in terms of not having the experience or support to deal with the situation]. The moral question in this case was formulated as: ‘Do I have to apply intensified supervision in this organisation or should I give a milder judgement so that she gets away with a progress report?’”

Values in cases in this theme that were mentioned by the participants were fairness: “Is it fair to punish a benevolent professional that has little debt to the organisation’s issues?” and decency “When do you exceed the boundaries of decency in health care regulation? The last question is about how far you, as an organisation, can go in a certain situation. The cases and moral questions in this theme are about the grey area between immediate measures and mild judgement.

### Theme 4: Is it allowed to give substantive judgements on care/profession?

Six of the 69 of the cases were categorized under the theme substantive judgement on care/profession. This specific theme contains dilemmas that rise during a regular inspection visit. These dilemmas are about the tension between procedural review (provides the organisation health care according to the current legislation and regulations?) and the substantive review of the health care provided. This can be illustrated by one of the cases:

“While visiting a health care organisation I see that clients are treated in a certain way. It seems that the welfare of the client is not the primary focus, however according to legislation it is permissible. A few months later I am at a different organisation that treats their clients from a very different care vision and this also works”. The moral questions for this inspector were: “Should I give a substantive judgement on the quality of health care?” and “May I express my preference for a care vision or must I stick to procedural judgement?”

From this case there seems to be a discrepancy between the used instruments in regulation and the opinion of the inspector. In one of the cases the inspector had a structural difficulty with a certain treatment of clients. Other examples of moral questions in this theme were: “Should I tell employees of an organisation that their manager makes the wrong choices?” and “May I refuse to give my opinion on substantive matters?” Important values that were mentioned in these last cases were: decency and carefulness (i.e. giving the right information to the organisation and collect enough information in an investigation before making a judgement).

### Theme 5: What is an appropriate role of the inspector (IGZ) in a conflict between other parties?

Because the inspectorate operates in a dynamic field with several stakeholders the inspectors are regularly involved in a conflict between two other parties. Six of the 69 of the analysed cases were categorized in the fifth theme: the role of the inspector in a conflict between other (third) parties. These parties can be clients and health care organisations, but also board of directors and professionals of different health care organisations or even professionals of the same organisation. One of the casus in this theme:

“I [the inspector] privately got information from a suspended director of a healthcare institution, which I had not received from the board of trustees before I visited the organisation, that was very important for the continuity of the organisation and patient care”. Two moral questions facing this inspector in this case were: “Can we allow that the IGZ is used by the differing parties to support their positions in a conflict?” and “May I use all information at a regulation visit that is entrusted to me?”

In the described case both the inactive board member and the board of trustees call the inspector with information about the other. The inspector has to decide what position IGZ has to take in this conflict but also to what extent he should consider the privately given information in the judgement. Other ethical challenges in this theme were: “Am I allowed to take information, that I received from another professional which blackens the name of a colleague, with a grain of salt?” In this specific case the inspector had difficulties in weighing up the received information because the decision to close the institutions would mean that a considerable number of patients would not receive the needed care.

### Theme 6: What are the boundaries of the professional responsibility?

Six of the 69 of the cases are categorized in the theme boundaries of the profession. The cases in this theme are about what does and what doesn’t fall under the responsibility of the inspector (or the IGZ in general). This is illustrated by the following case:

“A health care organisation has decided to no longer treat a certain group of patients. These patients are referred to two other health care organisations. However, this referral process proceeds badly. I (the inspector) am approached by the chairman of the patient association with the request to share some critical information on this matter with them”. The moral question of the inspector was: “Am I allowed to share this information?”.

In this case the inspector is confronted with the boundaries of the profession; is it my duty to inform the patient association about the bad referral process? Other examples of moral questions within this theme were: “Must IGZ initiate action in response to a publication in a newspaper showing that a health care giver exhibits transgressive behaviour?” and “A personal friend asks information on the quality of a certain caregiver for private reasons: may I disclose what I know about this caregiver?”.

In another case the inspector was asked by an external organisation to visit a specific organisation; is this allowed since it is not the normal procedure? In most of the cases the inspector is asked to share information or to take action in situations where it is unclear if it is the inspector’s job.

### Theme 7: Should we always adjust to the new and stricter IGZ policy?

When the pilot was conducted the IGZ introduced a new and stricter policy to the employees, in short it was expected of the inspectors that they enforced stricter. This change in policy caused some moral questions about their work. A total of 6 of 69 the cases are about this new (stricter) policy of the IGZ. The following case is an illustration of this theme:

“A professional is suspected of transgressive behaviour towards children. The investigation is still in progress and a disciplinary measure is considered. The suspicion is serious and the inspection has imposed a ban on working with children for this employee during the investigation. According to the new policy, the IGZ must publish the current investigation and the ban on working with children on their website, including name and address of the employee in question”. The moral question the inspector struggled with was: “Do we have to apply this new policy?”

Other moral questions regarding the new policy of IGZ were: “Several aspects of my current work which I value, are no longer covered by the vision of the government and the new policy of IGZ, am I still allowed to perform these activities?” and “May I, during a disciplinary measure, take into account extenuating circumstances or must I necessarily act on the new policy?” Values and norms that were discussed were amongst others: ‘one’s own integrity’ (i.e. as professional it is important that my actions are in line with my personal values); ‘justice’ (human level) and ‘loyalty’ (with respect to the new IGZ policy). Most cases are about how the inspector must adjust to the new policy.

### Theme 8: If and when do we have to share information?

The last analysed theme is about privacy and duty of confidentiality. Five of 69 of the cases are categorized under this theme. Whether or not to provide information is also mentioned in the theme boundaries of the profession. The cases under this theme differ in such a way that the situation in which it takes place actually is part of the profession.

An example of a case in this theme is:

“Several charges of sexual intimidation by a professional are being investigated. He is temporarily relieved of his duties and subsequently fired. Coincidentally I heard that this professional has applied for a position at another health care organisation”. The moral question of the inspector was: “Should I warn this new health care organisation?” Values and norms that were mentioned by participants as important in this case were: ‘patient safety’ (i.e. to avoid sexual intimidation) and ‘privacy of the professional’.

Other moral questions that were raised with respect to this theme were: “Do we have to report something without sufficient evidence?”, “Must I be silent against third parties on separate incidents when there is not yet any policy conclusion drawn?” and “To which extend can we (at IGZ) use names in the internal communication?”. Most cases in this theme concern the balance between protecting the health care organisation/professional and protecting patient safety.

#### Discussed cases

In every session the inspectors chose a case to discuss in the MCD and therefore eight of these ethical challenges (cases) were discussed in the MCD sessions. Table 2 6 shows in which of the themes the chosen cases were categorized. The distribution of the discussed casus seems to be equally divided over the themes. The high number in cooperation within the IGZ can be explained by the fact that it often joined another theme in a case.

**Table 2:** Number of cases discussed during MCD

Theme	Number of cases discussed in MCD
How should we relate to others?	2
How should we cooperate with each other within the IGZ?	5
Intensified supervision or equity?	2
Is it allowed to give substantive judgements on care/profession?	2
What is an appropriate role of the inspector (IGZ) in a conflict between other parties?	–
What are the boundaries of the professional responsibility?	–
Should we always adjust to the new and stricter IGZ policy?	1
If and when do we have to share information?	1

## Discussion

In this paper we described a thematic analysis of cases from a pilot study in which moral case deliberation (MCD) has been introduced in the Dutch Health Care Inspectorate (IGZ) in order to support the IGZ employees in dealing with their ethical challenges when monitoring and regulating the quality of health care. This paper presented the results of 69 ethical challenges, which the participating health care inspectors encounter during their daily job.

We identified eight themes in these ethical challenges. Inspectors of the Dutch health care inspectorate encounter ethical challenges in how they relate to others, in cooperation within the IGZ, in the debate between intensified supervision or equity, on the substantive judgement of care, on the role of the inspector (or IGZ) in a conflict between other parties, on the boundaries of the inspector’s profession, on the new and stricter policy and regarding privacy and the duty of confidentiality.

Two categories can be distinguished in the eight themes. The first category is the ethical aspect of *work content*. A majority of cases contains moral questions regarding the content of the profession of a health care inspector. How do I weigh certain information? When do I take the circumstances into account? What is good health care regulation?

The second category is the *internal collaboration*. How do we interact with colleagues? When do we address a colleague’s behaviour? Aligning continuously with colleagues (how do we relate to others and cooperation within the IGZ) seems necessary for proper health care regulation (Seekles et al. Seitzer). We presume that aligning between colleagues or program’s (departments) by means of MCD can contribute to a better inter-inspectors reliability or indicating a lack of it. A study of Tuijn et al. (2009) shows a large variation in judgements by inspectors. Working on better consistency and an increased inter-inspectors reliability starts with understanding the variation and building on a substantive support of the desired consistency. MCD does not primarily aim at reducing the variation between perspectives and opinions, but it generates more understanding of how colleagues perceive and reason in specific situations. Because these aspects are made explicit in MCD, it creates more grip on causes of variations in judgements and therefore on opportunities to reduce them. This might contribute to better quality of health care regulation. Studies on the role of MCD in health care (Molewijk et al. 2008b; Janssens et al. 2015) indicate that the quality of care is enhanced by reflection and dialogue. This might as indicated, also be true for health care regulation (see also Seekles et al. 2016).

### Themes in ethical challenges

In a study on moral issues in elderly health care institutions of Van der Dam et al. (2012) they found that most issues concerned, as they call it, the primary process (i.e. “What is good care for residents?”). In addition to this main category the professionals were also confronted with a small number of moral issues concerning the secondary care process (e.g. problems with distribution of shifts) (Van der Dam et al. 2012). They identify three themes in the primary process: resident’s behaviour, divergent perspectives on good care and organizational context. The divergent perspective is comparable with our theme substantive judgement on care. In both themes professionals struggle with their own opinion versus a different opinion (of a colleague, institution etc.) on what good care is. The theme organizational context in Van der Dam’s study (2012) contains work-content related issues such as restrictions of policy and lack of resources that may lead to less attention for a resident then desirable. The casuistry of our main themes, cooperation within the organisation seems in Van der Dam’s study to be distributed in both divergent perspective on care and organizational context. Another possible reason that in our study, compared to the one of Van der Dam (2012), a majority of cases considered internal collaboration issues might be that by the time we conducted this pilot study, the IGZ went trough a change of policy. Inspectors, some after years of working in regulation, had to adapt themselves to this new policy in which the IGZ is going to apply more stringent measures. We identified this as a separate theme, but the new policy might also be a cause of the adjustment that is necessary for a professional adapt to the renewed cooperation within an organisation.

### Ethical challenges in organisations

Comparable to the quality of care, we can conclude that the quality of supervision of health care is significantly morally loaded. Shale (2011) stated that improving health services in practice meant that many morally loaded decisions have to be made; questions of priority, standards, dissent and about what a reasonable compromise might be. These moral questions can cause uncertainty and doubt, but can also lead to disagreement between colleagues or between organisations which all can cause (moral) stress (Lützen et al. 2003). In a focus-group study on how health care professionals deal with ethical challenges, it appeared that many ethical challenges mentioned by these professionals were related to situations in which there was disagreement or conflict (Molewijk et al. 2015). Disagreements are inherent to differing perspectives and therefore in potential very useful for dialogue and reflexivity. However, not every team will equally constructive deal with these disagreements. West et al. (1997) found that reflexive teams show more detailed planning, pay more attention to long-term consequences and have a larger inventory of environmental cues to which they respond. Reflection and dialogue by means of clinical ethics support can help both health care professionals as inspectors deal with different viewpoints and situations in which disagreement or even conflict might come up.

### Future research

For future research it is recommended to investigate if the number of cases on organizational matters (collaboration and communication) decreases when professionals are supported in reflection and dialogue and become a more reflexive team. One of the hypotheses of the use of clinical ethics support is that employees and teams learn to deal with disagreement in a more constructive way.

### Ethics support within the Dutch Health Care Inspectorate

Based on the content and amount of casuistry we can conclude that the work of health care inspectors is morally loaded. Continuous judgements and important considerations regarding the quality of health care are made while choices have to be justified and substantiated. In addition the inspectorate and inspectors are constantly confronted with different parties and interests. The inspectorate is situated in a complex interaction between society, politics and media. This dynamic work environment asks a lot of the inspectorate staff regarding tact, weighing information, interpreting, analysing and justification of decisions. Evidence-based knowledge and rules or policy guidelines are only partially sufficient to deal with moral issues in concrete situations: in the end it also comes down to practical wisdom within the specific moment and a critical dialogue based on concrete experiences (Abma et al. 2009, 2010; Widdershoven and Molewijk 2010). The moral aspects of health care supervision presented in the cases, together with the urgency (Seekles et al. 2016) and need to deal with these questions in health care professions (Slowther et al. 2001), show that ethics support is necessary for health care inspectors. According to inspectors that participated in the MCD pilot (Seekles et al. 2016), it is important that ethics support (e.g. moral case deliberation) is integrated in policy and education programs of the organisation.

The importance of ethics support in health care regulation together with the earlier mentioned recommendation of ethics support by the Dutch ministry of health (VWS 2007) points out the need for implementing some form of ethics support in health care regulation. The Dutch minister of Defence wrote in February 2016 a letter to the House of Commons regarding integrity and stated that in order to keep Defence “moral fit” they developed moral teachings consultations and regularly organise moral case deliberation (Hennis 2016). Therefore we can assume that the need for ethics support applies to a wider area in governmental organizations than only the health care inspectorate.

## Conclusion

In this pilot study on moral case deliberation in the Health Inspectorate, we identified eight themes in the ethical challenges of the inspectors. These eight themes can be divided in two categories: work content related and internal cooperation. Moral issues are inherent to regulation, whether this is about supervision on health care, education or financial markets. Many of the dilemmas seem to be generic for the field of regulation. Based on the evaluation of our pilot study (Seekles et al. 2016) and the analysis of the casuistry, we advise the inspectorate to organize ethics support for its professionals and recommend that future research should examine both the relevance and the effects of ethics support on the quality of regulation outside the realm of health care.

## Footnotes

1 We use the term healthcare in a broad sense, which includes health care policy, processes, care and cure.

2 Other normative verbs could also be possible (e.g. must I, am I allowed to, what do I ought to do). Sometimes the case presenter even used two different verbs (e.g. Must I do A or am I allowed to do B?). The case presenter chose the normative verb on the basis of what fitted best.

3 Parts of this table are previously published in Dutch in Tijdschrift voor Toezicht (Seekles et al. 2015).

4 Cases in which the other is a colleague or a manager, the cases are categorized under the second theme (How should we cooperate with each other within the IGZ?).

5 LMO is a meeting of inspectors in which nationwide reports are discussed.

6 The content of this table is used in a larger table that is previously published in Dutch in Tijdschrift voor Toezicht (Seekles et al. 2015).
